# Case of Schirrus

**Published:** 1865-11

**Authors:** R. S. Lewis

**Affiliations:** Dubuque, Iowa


THE
### CHICAGO MEDICAL EXAMINER.

N. S. DAVIS, M.D., Editor.

VOL. VI. NOVEMBER, 1865. NO. 11.

### Original Contributions.

ARTICLE XXXVII.

### CASE OF SCHIRRUS.

By R. S. LEWIS, M.D., of Dubuque, Iowa.

Editor Chicago Medical Examiner,

*Dear Sir:*—If the following notes of a case, which came under my care, are worthy of a place in your valuable journal, you are at liberty to publish them. It has been a case of considerable interest to me, from its being so rare, and its occurring to a maiden lady. My opinion is, that it is a case of schirrus, from the tumor being hard and lobulated.

Oct. 9th.—Miss M——y, Irish, supposes her age to be 45 or 50 years, called to consult me for what she called falling of the womb; has been more or less troubled for two years past. She had walked five miles, from the country. I advised astringent applications, with the view of introducing a pessary.

Oct. 10th.—She attempted to walk back to the country, but the tumor had forced itself so far into the world that she was obliged to desist, and returned to the city, and called me to visit her. Found her suffering severe pain; and, on examination, I found the uterus protruding six inches beyond the vulva. I called Dr. W. Watson to see her with me, and, as she had not passed urine for 24 hours, we concluded to draw it off by catheter. In passing the catheter, she strained slightly, and forced the whole of the uterus into the world. About 9 P.M., Dr. WATSON armed a needle with a double ligature of strong silk twist, and thrust it through the neck of the tumor, tying it as tight as he could draw it on both sides. Some feeling of faintness came on, but soon passed off. The tumor was perfectly insensible, and the patient experienced no pain in passing the ligature through it; and the only inconvenience was its weight, producing a dragging sensation. Has experienced more or less uneasiness from it for two years past, and of late has been down occasionally, but has been easily returned into the vagina. Had no difficulty in passing the finger into a cul-de-sac, which terminated abruptly, after the tumor had come down. The neck of the uterus, where the ligature was passed, did not exceed an inch through.

Oct. 11th.—Had slept ordinarily well during the night; suffered no pain; pulse feeble, 100 per minute. Ordered pul. Dov., 10 grs., every three hours. Called again towards evening. Had some vomiting, evidently the effects of the Dover's powder. Tightened the ligatures. After leaving, her friends removed her about a mile, to another part of the city, against positive orders.

Oct. 12th.—Tightened the ligatures. Feels easy; stomach quiet; passed urine freely, for the first time since the ligature was applied; pulse still feeble, 100 per minute. Ordered port wine. No tenderness over the abdomen. Called again, in the evening, and tightened the ligatures.

Oct. 13th, 8½ A.M.—Found the ligatures loose, and on drawing the one on the right side, it cut through and came away; drew the other tight, and cut away the mass, which measured 9 inches in length, and 5 inches through, in its largest part; lobulated, intensely hard. The patient expressed great satisfaction in being relieved, and turned on her side. Ordered ext. of bark and iron, dessert spoonful, every three hours, in a glass of port wine; also a liberal allowance of beef-tea.

Oct. 14th, 9 A.M.—Passed a comfortable night. Pulse 88 per minute. Ordered pil. blue mass and ext. colocynth, one every four hours, until the bowels are moved. Continue medicine as before; also beef-tea. The ligature had retracted within the vagina. Passes urine freely.

Oct. 15th.—Feels somewhat exhausted from the movements of the bowels. Ordered opi., grs. j, ipecac, grs. j, sub-car. soda, grs. ij, repeat every two hours if necessary, to quiet the bowels. Ligature came away; no discharge from the vagina; appetite improving. Continue medicine and wine.

Oct. 16th.—No particular change, except the skin is moist; feels stronger, sits up for half an hour at a time, without fatigue.

Oct. 17th.—Feels refreshed, from a good night's rest; appears cheerful; pulse 98 per minute. Cleanse the vagina with sol-chlorate potas., ʒj, aqua, ℥viij. Continue medicine and nourishment.

Oct. 18th, 19th, and 20th.—Improving rapidly from day to day; pulse 98 and soft; sits up; moves about her room.

Oct. 21st.—Discharged. Moved into the country, feeling well, except weak.

